# Eye-specific quantitative dynamic contrast-enhanced MRI analysis for patients with intraocular masses

**DOI:** 10.1007/s10334-021-00961-w

**Published:** 2021-10-13

**Authors:** Myriam G. Jaarsma-Coes, Teresa A. Ferreira, Petra J. van Houdt, Uulke A. van der Heide, Gregorius P. M. Luyten, Jan-Willem M. Beenakker

**Affiliations:** 1 Department of Ophthalmology, Leiden University Medical Centre, P.O. 9600, 2300 RC Leiden, The Netherlands; 2 Department of Radiology, Leiden University Medical Centre, Leiden, The Netherlands; 3 Department of Radiation Oncology, The Netherlands Cancer Institute, Amsterdam, The Netherlands; 4 Department of Radiotherapy, Leiden University Medical Centre, Leiden, The Netherlands

**Keywords:** Dynamic contrast enhanced, Magnetic resonance imaging, Uveal melanoma, Pharmacokinetic modelling, Eye diseases

## Abstract

**Objective:**

Dynamic contrast enhanced (DCE)-MRI is currently not generally used for intraocular masses as lesions are small, have an inhomogeneous T_1_ and the eye is prone to motion. The aim of this paper is to address these eye-specific challenges, enabling accurate ocular DCE-MRI.

**Materials & methods:**

DCE-MRI of 19 uveal melanoma (UM) patients was acquired using a fat-suppressed 3D spoiled gradient echo sequence with TWIST (time-resolved angiography with stochastic trajectories sequence). The analysis consisted of a two-step registration method to correct for both head and eye motion. A T_1_ map was calculated to convert signal intensities to concentrations. Subsequently, the Tofts model was fitted voxel wise to obtain *K*^trans^ and *v*_e_.

**Results:**

Registration significantly improved the concentration curve quality (*p* < 0.001). The T_1_ of melanotic lesions was significantly lower than amelanotic lesions (888 ms vs 1350 ms, *p* = 0.03). The average achieved B_1_^+^ in the lesions was 91%. The average *K*^trans^ was 0.46 min^−1^ (range 0.13–1.0) and the average *v*_e_ was 0.22 (range 0.10–0.51).

**Conclusion:**

Using this eye-specific analysis, DCE of intraocular masses is possible which might aid in the diagnosis, prognosis and follow-up of UM.

**Supplementary Information:**

The online version contains supplementary material available at 10.1007/s10334-021-00961-w.

## Introduction

**Fig. 1:**
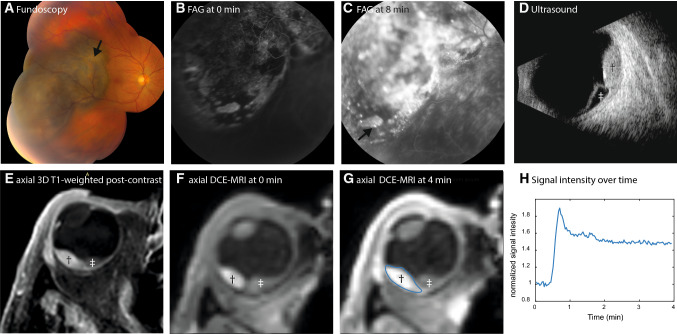
Conventional ophthalmic imaging and MRI of patient 13. **A**–**D** Conventional ophthalmic imaging of an UM. The fundus photo (**A**) shows a pigmented lesion with lipofuscin (arrow). The lesion is enhancing with pinpoints (**C**, arrow) on the fluorescent angiography (FAG, **B**, **C**). On ultrasound (US, **D**), the lesion (dagger) has an intermediate reflectivity, while the retinal detachment (double dagger) has a low reflectivity and the dimensions of the lesion are measured. On MRI, an enhancing lesion (dagger) with associated retinal detachment (double dagger) is visible (**E**–**G**). In contrast to the FAG, the change of the signal intensity after contrast administration can be visualized in DCE-MRI (**H**)

Most intraocular lesions are benign, such as choroidal neavi, haemangiomas and leiomyomas, but also various malignant intraocular masses exist. Although uveal melanoma (UM) is relatively rare, with an incidence of four to six per million in central Europe [1], it is the most common primary intraocular tumour. It mostly originates from the choroid (90%), but can also originate from the iris or ciliary body [2, 3]. Other malignant ocular lesions include mainly metastases from other tumour sites or even more rare lesions such as retinoblastoma [4, 5]. As the prognosis and treatment of benign lesions, UM and other malignant ocular lesions differ, it is important to have an accurate diagnosis [2, 5]. For the differentiation between these different lesions the ophthalmologist primarily relies on fundoscopic, fluorescent angiography and ultrasound imaging (Fig. 1a–d) [6]. However, in some patients, this differentiation is quite challenging, especially for amelanotic melanomas, or lesions behind the iris.

In the last decade, advances in ocular MRI, such as dedicated receive coils [7] and dedicated acquisition strategies [8] have resulted in different new clinical applications of MRI for ocular conditions [9]. MRI offers a superior evaluation of the extend of eye lid tumours [10], can be instrumental in the diagnosis and assessment of disease progression in orbital disease involving extra-ocular muscles [11], provide insight into ocular complaints such as negative dysphotopsia [12] and allows for a more accurate assessment of tumour dimensions for radiotherapy therapy planning [13, 14]. Furthermore, diffusion-weighted imaging is emerging as a promising early marker of therapy response after ocular proton-beam therapy [15], while quantitative dynamic contrast-enhanced MRI (DCE-MRI) could assist in the differential diagnosis of intraocular masses and monitoring of treatment response of UM [16]. The use of DCE-MRI of ocular masses is often limited to the evaluation of the time intensity curve (TIC, Fig. 1f–h) [16–19]. However, Wei et al. [17] and Kamrava et al. [20] have shown contradicting results on the relation between tumour permeability and metastatic risk. Kamrava et al. [20] found a higher *K*^trans^ in UM patients with monosomy 3, a subset of UM patients who have a strong increased risk of developing metastatic disease [21]. Conversely Wei et al. [17] showed a decreased *K*^trans^ (a lower peak signal intensity) in patients with metastatic disease. Although these papers are an improvement compared to the current clinical practice and other research where some eye-related challenges were not addressed such as small lesion size, eye motion and difference in melanin content.

The limited use of quantitative DCE-MRI might be due to the eye-specific challenges of MR imaging in general. One of the main challenges of DCE-MRI of intraocular masses is the small size of the eye, containing even smaller lesions, generally with a thickness of less than 5 mm. Furthermore, the eye is prone to movement, which in combination with the small lesion size leads to a mismatch of the tumour location between timepoints [18]. Finally, intraocular lesions can be pigmented (large amount of melanin), unpigmented (no melanin) or partially pigmented [22]. This varying degree of pigmentation results in a large variability in pre-contrast longitudinal relaxation time (T_1_) [23], which directly affects the quantification of concentration of the, generally T_1_-based, contrast agent. However, recent improvements in ocular MR-imaging protocols such as the use of a surface coil for receiving the signal and implementation of time-resolved angiography with stochastic trajectories sequence [24] allow for the acquisition of DCE-MRI with sufficient temporal and spatial resolution to perform DCE-MRI [18]. The aim of this paper is to overcome eye-specific challenges in the DCE-MRI analysis of intraocular masses.

## Methods

### Study population

Nineteen patients diagnosed with UM were included for this study. Nine patients were scanned as part of a prospective study and were recruited randomly. This study has been approved by the local ethics committee and subjects were scanned after written informed consent. The data from the remaining ten patients were selected from UM patients with a tumour prominence > 3 mm who received an MRI as part of clinical care. This retrospective inclusion of data was approved by the local ethics committee. The patients were on average 63 years old (range 30–82 years), 68% (*n* = 13) were male and had a BWI of 26.6 ± 4.5. The lesions had an average prominence of 7.8 mm and an average largest basal diameter of 14.5 mm on ultrasound. The American Joint Committee on Cancer staging [25] ranged from T1 to T4 with most patients in the T3 (*n* = 8) and T4 (*n* = 6) stage. Most were primary tumours (18/19) but in one case (UM15), the patient had a large reoccurrence. The primary tumour was treated with ruthenium plaque therapy and was located at the other side of the eye. A detailed description of the cohort of patients can be found in Table 1.

**Table 1:** Patient characteristics

Patient no	Tumour stage	Age at diagnosis (years)	BMI	Prominence on US (mm)	Largest basal diameter on US (mm)
UM 1	T3a	71	20	3.3	14.9
UM 2	T3a	61	30	12.3	14.4
UM 3	T2c	82	Missing	4	10.5
UM 4	T2b	68	22	5	11
UM 5	T4a	71	29	7.3	15.7
UM 6	T2a	73	32	2.8	9.5
UM 7	T3b	30	22	6.1	14.9
UM 8	T3a	37	35	6	14.6
UM 9	T2a	50	29	2.5	10
UM 10	T4a	80	27	12.1	15.5
UM 11	T1c	73	28	5.8	6.6
UM 12	T4a	62	19	11	22.6
UM 13	T4a	59	31	5.7	17.4
UM 14	T4b	75	26	13.9	18.8
UM 15	T3b	83	26	9.5	15
UM 16	T3b	64	21	8.4	15.6
UM 17	T4b	45	27	13	18
UM 18	T3b	65	29	9.1	14.8
UM 19	T3a	53	25	9	15.1

*US* Ultrasound

### MRI protocol

**Fig. 2:**
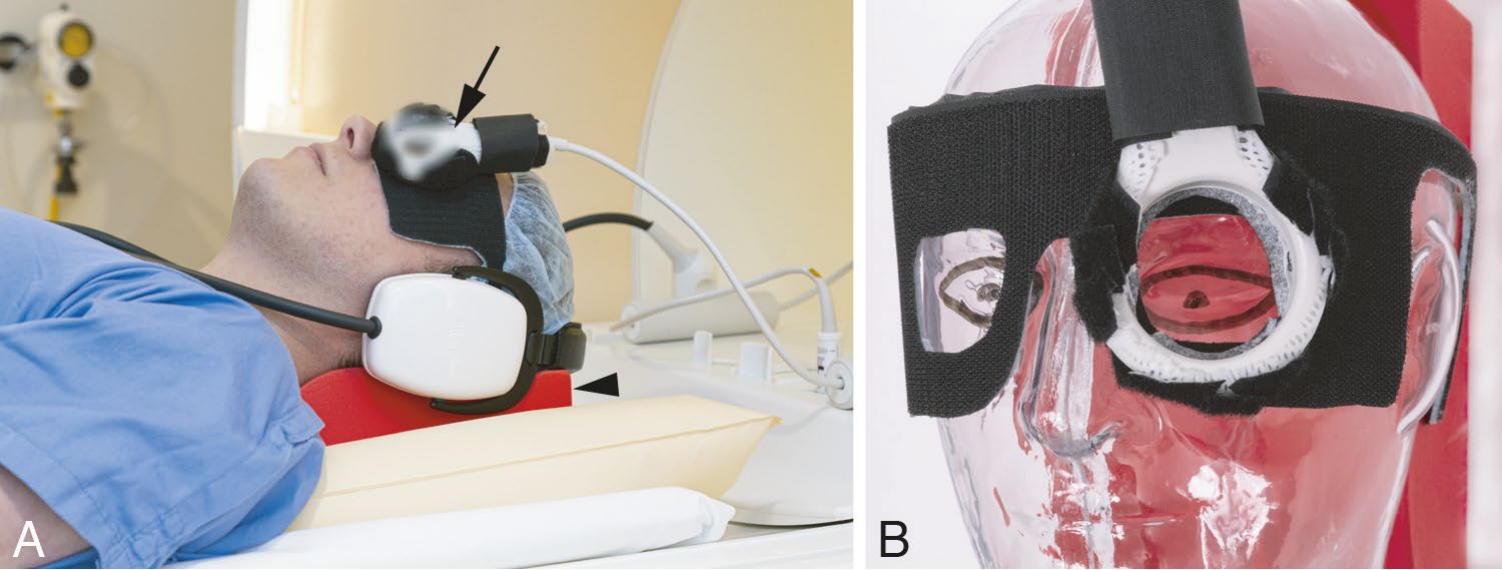
Clinical setup for ocular MRI. **A** The patient was scanned using a 47 mm surface receive coil (arrow). This coil was positioned over the affected eye. The head was supported by a radiotherapy head support (arrowhead). **B** Clarification of the positioning of the eye coil on a phantom

All patients were scanned before treatment on a 3T MR scanner (Ingenia, Philips Healthcare, the Netherlands) using the protocol described by Ferreira et al. [18] and a 47 mm diameter surface receive coil covering the affected eye (Fig. 2). The scan parameters of the relevant sequences are listed in Table 2. Patients were instructed not to wear eye makeup and the affected eye was taped shut. In the last six patients, a wet gauze was placed on top of the eye to minimize susceptibility artefacts. Patients were immobilized as much as possible using a radiotherapy head support (MaxSupport™ wide shaped, red variant, 117,000 HSSETW, Medeo, Schöftland, Switzerland) (Fig. 2).

**Table 2:** Scan parameters

	T_1_-mapping	B_1_^+^-mapping	DCE-MRI	3D T_1_-weighted
Voxel size (mm^3^)	1.25 × 1.5 × 1.5	2.0 × 2.0 × 2.0	1.25 × 1.5 × 1.5	1.0 × 1.1 × 1.0
FOV (mm^3^)	80 × 80 × 32	160 × 120 × 33	80 × 80 × 32	80 × 80 × 40
TR(ms)	7	7.1	4.5	350
TE(ms)/TE2(ms)	3.1	4.6/6.9	2.3	9.4
FA(deg)	2/5/9/15	10	5 or 13	90
Fat. Sup	Proset 11	SPIR	Proset 11	SPIR
Averages	1	2	1	1
Scantime (mm:ss)	4 × 00:09	00:21	04:20	03:23
Remarks		0.2 mm gap	2 s/dynamic; TWIST	After contrast

*FOV* Field of view, *TR* repetition time, *TE* echo time, *FA* flip angle, *Fat.Sup* fat suppression, *SPIR* Spectral Presaturation with Inversion Recovery, *TWIST* [24] time-resolved angiography with stochastic trajectories sequence, a dynamic scan technique where the a semi-randomly part of the 20% of outer *k*-space is acquired per dynamic

The dynamic time series were acquired using a fat-suppressed 3D spoiled gradient echo sequence with a spatial resolution of 1.25 × 1.5 × 1.5 mm^3^. A bolus of 0.1 mmol/kg gadolinium (DOTAREM; Guerbet, Roissy CdG Cedex, France) was administered 6 s after the start of the scan followed by a 20 ml injection of isotonic saline, using a power injector with an injection rate of 2 ml/s. The first eight patients were scanned with a flip angle of 5° to match the then used 7 Tesla protocol, where the flip angle was limited to 5° due to SAR restrictions. The flip angle was increased to 13° for subsequent patients as this provided a more optimal contrast for the contrast agent concentrations in our patients as theoretically the optimal flip angle for our spoiled gradient echo sequence is between 13° and 16° assuming a T_1_ between 600 and 1500 ms [26]. Time-resolved angiography with stochastic trajectories sequence (TWIST) [24], with a central size of 25% and a peripheral density of 20%, was implemented to increase the temporal resolution to 2 s per dynamic scan to reduce motion artefacts per dynamic image. To determine the baseline lesion T_1_, a 3D spoiled gradient echo flip angle series, with flip angles of 2°, 5°, 9° and 15°, was acquired before the dynamic scan with the same field of view (FOV). Additionally, a B_1_^+^-map was acquired using the DREAM sequence [27]. Finally a post-contrast 3DT1-weighted scan (3DT1gd) was acquired for anatomical reference.

### DCE analysis

The analysis of the DCE data consisted of four steps. First, the images of the dynamic scan, flip angle series, B_1_^+^-map and 3DT1gd scan were registered to the dynamic dataset and the lesion was segmented on the 3DT1gd image. Second, a T_1_ map was calculated using the flip angle series and B_1_^+^-map, which was subsequently used to calculate the gadolinium concentration for each lesion voxel in the dynamic scan. Finally, pharmacokinetic modelling was applied using the Tofts model [28].

#### Registration

All timepoints of the dynamic scan were rigidly registered to the 50th of the 125 timepoints in two steps using Elastix 4.9.0 [29]. The 50th timepoint was chosen as it had an intensity comparable to most timepoints, which was beneficial for automatic image registration. The first step consisted of registration of the full FOV to correct for head motion. Subsequently an eye-mask was created using an in-house build Mevislab network (3.0.2, MeVis Medical Solutions AG, Bremen, Germany [30]). The eye-mask was used for a masked registration to reduce eye motion between timepoints. Additionally, the variable flip angle series, B_1_^+^-map and 3DT1gd scan were rigidly registered to the 50th timepoint using masked registration.

#### Segmentation

A lesion mask was created by manually segmenting the UM on the 3DT1gd images using ITK-SNAP [31]. Elastix was used to translate this mask to the registered dynamic scan, using the earlier obtained transformation matrix. Subsequently, the voxels within this mask were selected for the pharmacokinetic analysis.

#### T_1_-mapping

The pre-contrast T_1_ value of each voxel was obtained from the flip angle series in Matlab (version R2019b, MathWorks, Natick, Massachusetts, USA) as described by Fram et al. and Gupta et al. [32, 33]. The flip angles were corrected according to the median achieved B_1_^+^ of the lesion. Subsequently, a masked 3D median filter with 26-connected components was applied to the T_1_ map to remove potential outliers within the lesion. Voxels outside the lesion were excluded from the filter, as the vitreous has significantly higher T_1_ values than the lesion [34]. The UM were classified as melanotic, amelanotic or mixed based on description of the tumour in the medical status by an ophthalmologist.

#### Pharmacokinetic modelling

The signal intensities from the DCE images were converted to concentration time profiles, using the relations described by Tofts [28], assuming a gadolinium relaxivity of 3.4 L mmol^−1^ s^−1^ [35]. For each voxel, the peak concentration was defined as the 95th percentile of the concentration over time.

Voxel-by-voxel pharmacokinetic modelling (PKM) was performed using nonlinear least squares fitting of the standard Tofts model using in-house build scripts in Matlab. First, the bolus arrival time (BAT) was determined for each patient by fitting the PKM for the first 40 time points of the median lesion concentration curve for 25 different BATs. The BAT with the lowest residuals was selected. The automatic demined BAT was visually correct in 50% of the patients. In the remaining patients, the BAT was shifted with one timepoint in eight cases and two timepoints in three cases. Subsequently, the PKM was fitted to the full dynamic concentration curve (*C*(t)) for each voxel within the lesion to obtain the *K*^trans^ (vascular permeability [min^−1^]) and *v*_e_ (extravascular extracellular space per volume of tissue [unitless]). As no major feeding arteries were in the field of view of the DCE scan, an earlier derived population arterial input function (AIF) was used, which was derived from the carotid arteries in ten brain cancer patients (supplementary Fig. 1)*.*

#### Evaluation of the registration

The effect of the registration was evaluated by comparing the concentration curves before and after registration of 15 randomly selected lesion voxels for all patients. The curves from all patients were randomized and presented unannotated to prevent a potential bias. The observer scored each curve as being sufficient or insufficient for automatic fitting and scored which of the two curves had the best quality or whether the quality was the same based on the amount of visual variance/spikes and motion artefacts in the concentration curve. Observer 1 (MJ) scored all 285 curves and observer 2 (JWB) scored a random subset of 50 to validate the scores. The scores were evaluated using a two-sided Wilcoxon signed rank test.

#### Evaluation of the error propagation

The effect of the precision error of the B_1_^+^, T_1_ and registration on the pharmacokinetic were assessed. First, in two additional UM patients, the flip angle series and B_1_^+^ map were acquired twice to determine the repeatability of the B_1_^+^ and T_1_ measurements. Second, the effect precision errors in B_1_^+^, T_1_ and registration on *K*^trans^ and *v*_e_ was assessed to determine the sensitivity of small inaccuracies of the different analysis steps on the final DCE parameters. To this end, the results of different intermediate steps were manually modified in two different patients: a patient with a medium-sized amelanotic tumour with a B_1_^+^ of 82% (patient 7) and with a large melanotic tumour and a B_1_^+^ of 96% (patient 10). The effect of a precision error in B_1_^+^ measurement was assessed by increasing and decreasing the measured B_1_^+^ with 2 and 5%. The sensitivity to T_1_ changes was determined by changing the measured T_1_ for all voxels by 30 ms, the measured precision error for the T_1_ mapping, or 60 ms, and 2 and 5% of the average tumour T_1_, respectively. Finally, the effect of imprecise registration was estimated by artificially shifting the images of individual timepoints. Regular eye motion was assessed by a one voxel shift during 2 timepoints after 175 s, while the most unfavorable case was assessed by the same shift but exactly after bolus arrival. For all cases, the median K^trans^ and *v*_e_ of the tumor were compared with the original analyses.

#### Statistical analysis

The impact of the different analysis steps on the final PKM was evaluated. The *K*^trans^ values with and without registration and with and without B_1_^+^-correction were visualized. The effect of T_1_-mapping was evaluated by comparing the *K*^trans^ values based on concentration data with the average T_1_ of all patients with the model in which data from the individual T_1_ map was used. The difference in *K*^trans^ between melanotic and amelanotic lesions for a population T_1_ and individual T_1_ was tested using unpaired *t* tests.

The reported PKM values in this paper are the median of the voxels within the lesion mask as the values are not normally distributed. The error bars shown in the figures are the 25th and 75th percentile (IQR). The reported unpaired *t* tests were calculated using Matlab. A *p* value of 0.05 or smaller was considered statistically significant.

## Results

### Registration

The centre of the eye moved up to 3.0 mm (average 1.3 mm) during the 4 min acquisition of the dynamic time series with rotations of up to 20° (average 6°) with respect to the first time point.

**Fig. 3:**
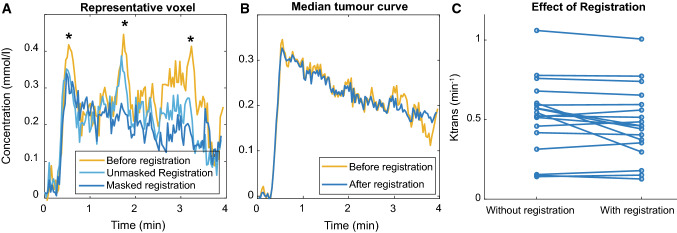
Registration. **A**, **B** Concentration time curves of a representative patient (18) before and after registration. Before registration motion artefacts were present (asterisk); furthermore, unmasked registration was not able to fully resolve the motion. A movie of the dynamic scan of patient 18 visualizing the motion in the scan can be found in the supplementary materials. **C** Registration changes the *K*^trans^ of the UM patients with an average of 0.06 min^−1^

Registration resulted in a significant improvement in the quality of the concentration curves (*Z* = 8.9, *p* < 0.001). Figure 3 shows a representative curve before and after registration. Eye motion can result in changes in the enhancement and concentration curves, as even small eye motion can result in mismatch between the ROI and actual lesion location, as can be seen in Fig. 3. Without registration, there were spikes (Fig. 3, asterisk) in the contrast agent concentration, which were caused by motion, most likely eye blinks or a different gaze angle. In this case, most of the outflow was no longer apparent after registration of the eye, although still some residual motion artefacts can be seen in the concentration curve. Note that eye motion was not resolved with the unmasked registration.

Before registration, 40% (*n* = 113/285) of the curves were scored as insufficient quality to perform a fit compared to 15% (*n* = 43/285) after registration. In 55% of the curves (*n* = 157/285), the curve was scored as improved, in 36% (*n* = 102/285) of the curves, no clear effect of the registration was observed, while in 9% (*n* = 26/285), the curve was scored as deteriorated after registration, although in 15/26 of these curves, the curves received the same quality, indicating a minimal difference. No significant difference in scoring was found between the scoring of observer 1 and 2 (*p* = 0.09).

The reported increase in quality of the concentration curves after registration resulted in a change of the PKM parameter values. The maximum absolute change in *K*^trans^ was 0.25 min^−1^ with an average absolute change of 0.06 min^−1^ (Fig. 3c; Table 3).

**Table 3:** *K*^trans^

Patient	*K*^trans^	Without registration	% change	Without B_1_^+^	% change	Average T_1_	% change
1	0.20	0.18	− 10	0.27	36	0.26	31
2	0.46	0.57	24	0.77	68	0.70	51
3	0.37	0.60	62	0.50	33	0.19	− 50
4	0.13	0.15	18	0.17	35	0.16	23
5	0.46	0.51	11	0.55	20	0.52	13
6	0.15	0.14	− 6	0.18	20	0.11	− 28
7	0.30	0.54	84	0.44	49	0.53	80
8	0.40	0.42	3	0.50	24	0.55	35
9	0.35	0.32	− 11	0.44	24	0.52	47
10	0.77	0.78	1	0.85	9	0.94	22
11	0.18	0.15	− 14	0.29	64	0.19	7
12	0.44	0.57	29	0.44	0	0.43	− 2
13	0.65	0.68	4	0.76	16	0.46	− 30
14	0.51	0.52	2	0.72	39	0.29	− 44
15	0.59	0.59	− 1	0.80	35	0.27	− 55
16	0.49	0.46	− 6	0.61	26	0.20	− 59
17	1.01	1.06	5	1.03	3	1.17	17
18	0.56	0.52	− 7	0.68	21	0.25	− 55
19	0.74	0.76	3	0.70	− 6	0.82	10

### B_1_^+^ and T_1_-mapping

**Fig. 4:**
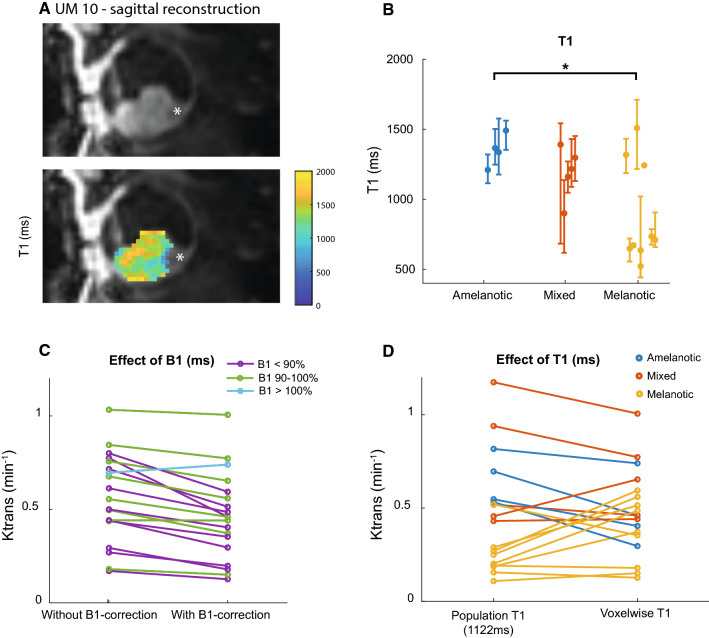
T_1_-mapping. **A** Slice of UM patient 10 showing an inhomogeneous T_1_ of the lesion. The retinal detachment (Asterix) is excluded from the analysis. **B** T_1_ values of amelanotic, mixed, melanotic and non-UM lesions (median and IQR). The three melanotic lesions with a higher T_1_ are small lesions most likely suffering from partial volume effects. The T_1_ between amelanotic and melanotic lesions is significantly different (*p* = 0.03). No significant difference was found between mixed lesions and either amelanotic or melanotic lesions. **B**, **C**. B_1_^+^-correction change the *K*^trans^ with an average of 0.11 min^−1^. **D** When a population T_1_ was used the *K*^trans^ mainly resembled the amount of pigmentation with a low *K*^trans^ for melanotic lesions. When the actual T_1_ was used the *K*^trans^ changed with an average of 0.15 min^−1^

The median lesion T_1_ per patient showed a wide range from 522 to 1509 ms as is shown in Fig. 4b. The average T_1_ of all patients was 1122 ms, this value was used as population T_1_ for subsequent comparisons on the effect of T_1_ on the PKM. Amelanotic lesions had an average T_1_ of 1350 ms, while the average T_1_ of 888 ms for the melanotic lesions was significantly lower (*p* = 0.03). The average T_1_ of the mixed lesions was 1193 ms.

The average achieved B_1_^+^ in the lesion was 91% (range 77–104%). Correction of the flip angles resulted in an average absolute change in T_1_ of 208 ms and an absolute change in *K*^trans^ of 0.11 min^−1^, (Fig. 4c; Table 3).

When a population average T_1_ was used the *K*^trans^ appeared to mainly resemble the amount of pigmentation, with the melanotic showing a significant lower *K*^trans^ compared to amelanotic lesions (*p* < 0.01, Fig. 4d). However, when the actual T_1_ was included in the analysis, the *K*^trans^ changed with 0.15 min^−1^ on average (Fig. 4d; Table 3) and the bias was resolved as no systematic difference was found between the *K*^trans^ of melanotic and amelanotic lesions (*p* = 0.37).

**Fig. 5:**
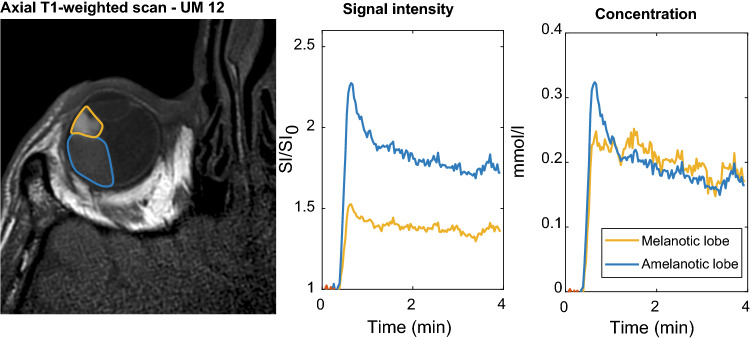
The effect of T_1_ on the signal intensity. Bilobar lesion with both an amelanotic and a melanotic lobe. The amelanotic part of the lesion appears to be enhancing stronger than the melanotic part. When, however, the actual T_1_ was included in the calculation of the contrast agent concentration, a very similar concentration was found in both lesions, although still a distinct difference was present in the early timepoints

The strong effect of the melanin concentration on the perfusion quantification can be seen in a mixed lesion with both a melanotic and amelanotic lobe, (Fig. 5). On the TIC, the amelanotic part of the lesion appeared to be enhancing stronger than the melanotic part, 225% vs 150%. The melanotic lobe has, however, an almost 1000 ms shorter T_1_ than the amelanotic lobe, on average 494 ms vs 1464 ms, respectively. When the T1 was included in the conversation to concentration, a very similar concentration was found in both lesions, although still a higher peak concentration was measured in the early timepoints of the amelanotic lobe.

### Pharmacokinetic parameters

**Fig. 6:**
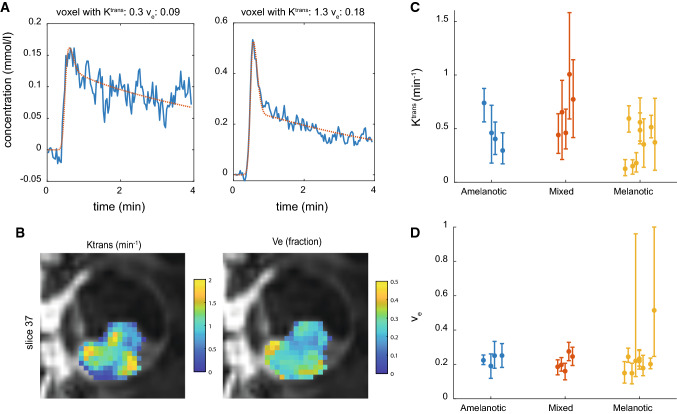
Pharmacokinetic modelling results. **A** Concentration curve and fit of two voxels of patient 10 showing the inhomogeneity of the lesion. **B** A sagittal slice of UM patient 10 showing *K*^trans^ and *v*_e_ maps showing an inhomogeneous values of *K*^trans^ and *v*_e_ in the lesion. **C**, **D** The median and IQR of the *K*^trans^ and *v*_e_ in the lesion. All patients except one UM had a *v*_e_ lower than 0.3

When all corrections were applied, a wide range of *K*^trans^ values was observed (Fig. 6c). The median *K*^trans^ per lesion ranged from 0.13 to 1.0 min^−1^ with a mean of 0.46 min^−1^. The median *v*_e_ was 0.22 on average with a range from 0.10 to 0.51.

Within lesions, a wide distribution of *K*^trans^ and *v*_e_ was observed. The maximum IQR (75th–25th percentile) of the *K*^trans^ was 0.99 min^−1^ and the average IQR was 0.40 min^−1^. For the *v*_e_, the maximum IQR was 0.83 and the average IQR was 0.18.

### Error propagation

The average precision error of the achieved B_1_^+^ and T_1_ was 0.2% and 30 ms, respectively. Major inaccuracies (5%) in the measurement of the achieved B_1_^+^ can result in a difference up to 12% in the determined *K*^trans^ and 19% in the determined *v*_e_ (Table 4). Differences in the T_1_ measurement (30 ms) can lead to changes in *K*^trans^ and *v*_e_ up to 5% (Table 4). Imprecisions in the registration during the second half of the acquisition had a minimal effect on the outcomes of *K*^trans^ and *v*_e_ (< 1%), while a similar imprecisions directly after contrast uptake resulted in 3% change in *K*^trans^.

**Table 4:** Error propagation

Sensitivity to inaccuracies in the registration	Favourable location		Unfavourable location						
**Relative change in**	*K*^trans^	*v*_e_	*K*^trans^	*v*_e_					
*Plateau curve/medium-sized UM*	− 0.5%	− 0.6%	3.0%	− 0.5%					
Washout curve/large UM	− 0.2%	0.1%	2.6%	0.1%					
Sensitivity to inaccuracies in B_1_^+^	B_1_^+^ − 5%		B_1_^+^ − 2%		B_1_^+^ + 2%			B_1_^+^ + 5%	
**Relative change in**	*K*^trans^	*v*_e_	*K*^trans^	*v*_e_	*K*^trans^	*v*_e_		*K*^trans^	*v*_e_
*Low achieved B*_*1*_* (82%)*	− 10.7%	− 7.2%	− 3.6%	0.3%	6.4%	10.6%		14.1%	18.7%
Normal achieved B_1_(96%)	− 11.8%	− 7.4%	− 5.8%	− 1.2%	2.5%	7.5%		8.9%	14.2%
Sensitivity to inaccuracies in T_1_	T_1_ − 60 ms		T_1_ − 30 ms		T_1_ + 30 ms			T_1_ + 60 ms	
**Relative change in**	*K*^trans^	*v*_e_	*K*^trans^	*v*_e_	*K*^trans^	*v*_e_		*K*^trans^	*v*_e_
*Amelanotic UM*	10.7%	9.4%	5.4%	4.8%	− 5.0%	− 4.7%		− 8.9%	− 9.2%
Melanotic UM	6.7%	6.0%	3.3%	3.1%	− 3.4%	− 2.8%		− 6.8%	− 5.8%

## Discussion

Recent developments in ocular MR imaging allow for the acquisition of DCE images with sufficient temporal and spatial resolution to perform DCE-MRI in the eye, as the currently achievable isotropic spatial resolution of ~ 1 mm is sufficient to assess the smaller intraocular lesions, while the 2 s temporal resolution yields more than sufficient time points to determine the inflow characteristics of the lesion [18]. With this improved protocol, DCE-MRI was performed in 19 intraocular lesions and the effect of eye-specific challenges on the quantification was investigated.

As the eye can rotate within the head, registering the complete FOV of all time points is not sufficient to correct for eye motion. Therefore, a dedicated registration method was developed to mitigate the effect of gazing variations on the measured concentration curve. These corrections resulted in absolute changes in *K*^trans^ up to 0.25 min^−1^. Although rigid registration of the complete Field-of-View has been proposed to correct for motion of intraocular lesions [20], we argue that is not sufficient, since the eye moves within the head. We showed that masked-registration improved the quality of the curves significantly, however, still the quality of 15% of the concentration curves was scored as insufficient to perform an automatic fit on. Smaller lesions had a higher percentage of insufficient quality voxels indicating that our registration cannot fully resolve effects of motion at the edge of the lesion. In 4% (*n* = 10/285) of the curves, across different subjects and tumour sizes, registration seemed to have deteriorated the quality at some timepoints, although the deterioration was minor compared to the improvement by the registration in the other voxels. This mostly occurred at the edge of the tumour and might be attributed to partial volume effects, although inhomogeneities in the tumour might play a role as well. A MRI protocol with an increased resolution might, therefore, also be favourable to decrease the effect of these residual errors in the registration. Although cued blinking might be implemented to reduce motion during acquisition, the resulting twofold reduction in temporal resolution will likely not be beneficial for the pharmacokinetic modelling [7, 8]. It might be beneficial to register all scans to the higher resolution 3DT1gd instead of one of the dynamic timepoints.

Only one paper [20] was found that used T_1_ mapping for the quantitative DCE analysis of ocular lesions. We found a significant difference in T_1_ between melanotic and amelanotic lesions and a strong effect on the quantification indicating that T_1_ mapping is a crucial step in DCE quantification. We, therefore, recommend to include the actual T_1_ in the analysis of DCE of ocular lesions to prevent the bias introduced by the amount of melanin in the tumour. The effect of melanin can be seen clearly in patients with a bilobar lesion, as shown in Fig. 5. A similar effect was observed between lesions, where amelanotic lesions appears to enhance more than melanotic lesions. This was, however, primarily the result of its longer T_1_ and not of an increased contrast agent concentration.

Finally, our results indicate that registration and B_1_^+^correction are important steps in the quantitative analysis of ocular DCE, but these steps affect the pharmacokinetic parameters to a lesser extent than the differences in T_1_, and are independent of the type of lesion. DCE scans are clinically often evaluated by assessing the Time Intensity Curves (TIC), instead of the actual gadolinium concentration which can be calculated from these intensities [16–19]. However, by comparing these TICs between lesions, the potential differences in T_1_ between these lesions are ignored, which can lead to erroneous interpretation of the data. A higher (maximum) relative enhancement or area under the curve might, for example, be interpreted as an increased perfusion in the lesion, but might actually be the result of a less pigmented lesion which has, therefore, a higher T_1_. As the overall shape of the TIC is not affected by the scaling effect of the T_1_, a classification based on the curve pattern can still be useful when only the signal intensity is available. [36, 37]

In line with earlier genetic and pathology studies [38–41], our results indicate that uveal melanomas are inhomogeneous, making a voxel wise volumetric assessment of ocular lesions preferred over a single 2D ROI analysis. The relatively thick slices of 3 mm used in the study of Kamrava, likely still resulted in representative sample of the lesion, due to averaging in the slice direction, resulting in the observed correlation between monosomy 3 and *K*^trans^. However, a lower resolution limits the possibility for motion correction and analysis of small intraocular lesions, making a higher resolution with a voxel wise analysis preferred. Overall, ROI-based (semi-)quantitative DCE-MRI analysis without T_1_ mapping for ocular lesions is not recommended as this would result in a significant bias as most DCE measures are dependent on the baseline T_1_.

There are areas in which ocular DCE can be further improved. First of all, the flip angle of the DCE-MRI acquisition was 5° for the first eight patients and 13° for subsequent patients. For the determination of reference PKM values of lesions, a standardized protocol should be used for all patients. On the hardware side, a multichannel eye coil [42, 43] might result in multiple advances in ocular DCE. An increased channel count might not only enable an increased temporal or spatial resolution, but also a potential larger FOV which can be used to determine the AIF on a patient-specific level. As with the single loop–coil approach no major blood vessels were available within the FOV, we relied on a population-based AIF. Population-based AIFs are a widely used approach but result a less accurate estimation of especially *K*^trans^ as the AIF is be influenced by body mass and cardiac output [44, 45]. Eye muscles were investigated as reference tissue, but the perfusion of the eye muscles appeared not to be consistent within a single subject and might be also influenced by lesion location and therefore unreliable.

Although the spatial and temporal resolution achieved with our protocol are high compared to other ocular DCE studies, the spatial resolution of 1.25 × 1.5 × 1.5 mm^3^ is still a limiting factor for small ocular lesions. The smallest lesion included in this study was 32 voxels. A higher resolution would be preferred as this allows for the edge voxels to be removed from the analysis, as the results from these voxels are less reliable due to partial volume effects, not only of the dynamic scan, but also in the T_1_ mapping. Therefore, for the current resolution, DCE-MRI of small lesions small lesions (i.e. thickness < 2 mm) is likely less accurate. In situations where the conditions are less optimal (e.g. no orbital coil is or only a 1.5T MRI is available), DCE-MRI of intraocular lesions could be performed, however, although the decrease in image resolution would increase the lesion size required for reliable results. Moreover, although the TWIST sequence successfully reduces the motion artefacts by reducing the acquisition time per dynamic, the effective spatial resolution for rapidly changing concentrations, in particular the inflow of the contrast agent, might be lower. Nevertheless, the effect of precision errors in analysis steps, such as T_1_ determination or registration, on the obtained pharmacokinetic parameters generally showed to be less than 5%. Furthermore, some choices in the proposed analysis pipeline might benefit from a more thorough evaluation to further improve the analysis, such as the optimal reference image for the registration, the potential benefit of applying the median filter to the source FA-series images instead of the resulting T_1_ map and other PK models, such as the extended Tofts model to include a vascular component [46]. Finally, it is important to assess the reproducibility of DCE-MRI for ocular lesions between visits and centers.

Some of the proposed steps to enable reliably ocular DCE-MRI, such as the eye-specific registration, might be less easily incorporated into clinical practice. However, the inclusion of differences in T_1_ between lesions in the analysis, which has the strongest effect on the PK measures, is available in various clinical software packages. Although the elementary TIC classification, e.g. a distinction between a washout and plateau curve, is not affected by the T_1_, other elementary measures, in particular the relative enhancement, are significantly affected. Clinically, this conversion to concentration is particularly important as conversely to UM, the majority of other intraocular lesions are non-pigmented, resulting a biased evaluation. When the pharmacokinetic parameters have been determined in a larger cohort of patients with intraocular lesions, they could aid to differentiate between benign and different malignant intraocular lesions. Although the eye-specific motion correction is currently not available clinically, head motion can still be corrected with regular registration methods. When only this form of registration is available, a careful evaluation of the individual data is needed to screen for motion and to potentially remove motion-corrupted time points. We anticipate that this approach will be clinically sufficient to aid in the differential diagnosis, especially as other information, such as DWI, can be included in the considerations. This research furthermore showed that some UM are inhomogeneous in composition. For subsequent studies to assess these inhomogeneities, and assess their potential relation to genetic factors and the patients’ prognosis [20], full motion correction will be needed, as these inhomogeneities can amplify the effect of eye motion on the final parameters.

## Conclusion

Although MRI of eyes is challenging in many aspects, we showed that quantitative DCE-MRI analysis can be performed for intraocular lesions by increasing the temporal and spatial resolution of the dynamic scan and using dedicated registration and T_1_ mapping with B_1_^+^ correction in the analysis. In the clinic DCE-MRI analysis might aid in the diagnosis, prognosis and follow-up of intraocular masses.

## Supplementary Information

Below is the link to the electronic supplementary material.

Supplementary file1 (AVI 12854 KB)

Supplementary file2 (DOCX 49 KB)
