# Supplementary material for: Eye-specific quantitative dynamic contrast-enhanced MRI analysis for patients with intraocular masses
Source: MAGMA. 2021 Oct 13;35(2):311–23. doi: 10.1007/s10334-021-00961-w (PMC8995252; doi:10.1007/s10334-021-00961-w)
Supplement: Supplementary file 2 — Supplementary file2 (DOCX 49 KB) [file 10334_2021_961_MOESM2_ESM.docx]

**Supplementary material**

**Supplemental table 1. Results per patient**

| **Patient** | **B_1_ tumour (%)**  **Median, 25^th^ & 75^th^ percentile** | | | **T1 tumour (ms)**  **Median, 25^th^ & 75^th^ percentile** | | | **Ktrans (min-1)**  **Median, 25^th^ & 75^th^ percentile** | | | **Ve (%)**  **Median, 25^th^ & 75^th^ percentile** | | |
| --- | --- | --- | --- | --- | --- | --- | --- | --- | --- | --- | --- | --- |
| UM 1 | 86 | 81 | 89 | 1300 | 1206 | 1368 | 0.20 | 0.10 | 0.33 | 0.10 | 0.07 | 0.15 |
| UM 2 | 77 | 74 | 81 | 1365 | 1248 | 1502 | 0.46 | 0.18 | 0.72 | 0.19 | 0.12 | 0.26 |
| UM 3 | 91 | 86 | 95 | 710 | 658 | 906 | 0.37 | 0.11 | 0.78 | 0.51 | 0.25 | 1.00 |
| UM 4 | 86 | 82 | 89 | 1318 | 1187 | 1432 | 0.13 | 0.06 | 0.21 | 0.15 | 0.09 | 0.22 |
| UM 5 | 91 | 89 | 92 | 1161 | 1047 | 1270 | 0.46 | 0.31 | 0.68 | 0.16 | 0.11 | 0.21 |
| UM 6 | 91 | 88 | 94 | 671 | 659 | 683 | 0.15 | 0.08 | 0.21 | 0.15 | 0.09 | 0.21 |
| UM 7 | 82 | 78 | 86 | 1491 | 1353 | 1562 | 0.30 | 0.17 | 0.46 | 0.25 | 0.18 | 0.32 |
| UM 8 | 90 | 88 | 92 | 1336 | 1176 | 1577 | 0.40 | 0.26 | 0.56 | 0.25 | 0.18 | 0.33 |
| UM 9 | 90 | 86 | 94 | 1243 | 1243 | 1243 | 0.35 | 0.14 | 0.59 | 0.18 | 0.13 | 0.25 |
| UM 10 | 96 | 94 | 97 | 1298 | 1130 | 1451 | 0.77 | 0.42 | 1.14 | 0.25 | 0.19 | 0.30 |
| UM 11 | 78 | 35 | 103 | 1509 | 1216 | 1711 | 0.18 | 0.10 | 0.28 | 0.22 | 0.13 | 0.96 |
| UM 12 | 100 | 96 | 103 | 1391 | 683 | 1543 | 0.44 | 0.27 | 0.64 | 0.19 | 0.14 | 0.23 |
| UM 13 | 93 | 86 | 97 | 900 | 618 | 1137 | 0.65 | 0.21 | 0.95 | 0.20 | 0.16 | 0.24 |
| UM 14 | 85 | 82 | 87 | 734 | 676 | 787 | 0.51 | 0.41 | 0.62 | 0.20 | 0.17 | 0.24 |
| UM 15 | 86 | 83 | 88 | 647 | 555 | 719 | 0.59 | 0.47 | 0.71 | 0.24 | 0.21 | 0.29 |
| UM 16 | 89 | 87 | 91 | 522 | 443 | 642 | 0.49 | 0.36 | 0.65 | 0.23 | 0.19 | 0.29 |
| UM 17 | 99 | 96 | 101 | 1217 | 1089 | 1430 | 1.01 | 0.59 | 1.58 | 0.28 | 0.22 | 0.33 |
| UM 18 | 91 | 87 | 95 | 636 | 441 | 1020 | 0.56 | 0.38 | 0.79 | 0.22 | 0.17 | 0.28 |
| UM 19 | 103 | 101 | 105 | 1210 | 1114 | 1320 | 0.74 | 0.56 | 0.87 | 0.22 | 0.20 | 0.25 |

**Supplemental figure 1. Parker AIF**

**
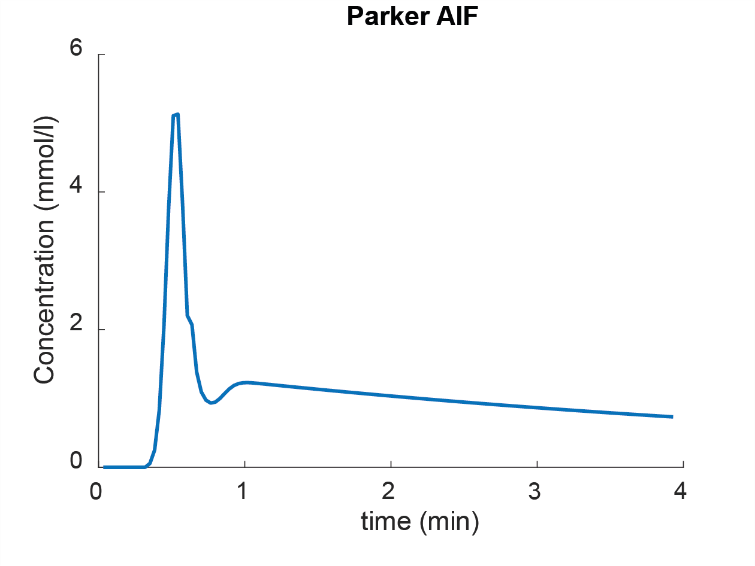
**The fit of the Parker AIF to the average AIF resulted in the following parameters: A1 = 46.7 mM∙s, σ1 = 3.5 s, T1 = 12.5 s, A2 = -4.7 mM∙s , σ2 = 5.2 s, T2 = 26.9 s, α = 1.4 mM, β = 0.003 s-1, s = 8.3 s-1, τ = 17.5 s.
